# Supplementary material for: Seroprevalence of anti-SARS-CoV-2 specific antibodies in vaccinated and vaccine naïve adult Nigerians
Source: PLoS One. 2023 Jan 23;18(1):e0280276. doi: 10.1371/journal.pone.0280276 (PMC9870169; doi:10.1371/journal.pone.0280276)
Supplement: Attachment — Submitted filename: Response to Reviewers.docx [file pone.0280276.s001.docx]

Dear Editor,

Kindly find below our responses to the comments raised by the Academic Editors and Reviewers.

Thank you for your consideration.

**Comment**

Thank you for submitting the above manuscript to PLOS ONE. During our internal evaluation of the manuscript, we found significant text overlap between your submission and previous work in the introduction. We would like to make you aware that copying extracts from previous publications, especially outside the methods section, word-for-word is unacceptable. In addition, the reproduction of text from published reports has implications for the copyright that may apply to the publications.
Please revise the manuscript to rephrase the duplicated text, cite your sources, and provide details as to how the current manuscript advances on previous work. Please note that further consideration is dependent on the submission of a manuscript that addresses these concerns about the overlap in text with published work.
We will carefully review your manuscript upon resubmission and further consideration of the manuscript is dependent on the text overlap being addressed in full. Please ensure that your revision is thorough as failure to address the concerns to our satisfaction may result in your submission not being considered further.
**Response**

The authors would like to thank you sincerely for this observation. The introduction section has been revised as suggested (revision carried out is as highlighted in the revised manuscript with track changes).

**Comment**

Has the statistical analysis been performed appropriately and rigorously?

Reviewer #1: No

Reviewer #2: No

**Response**

The authors would like to state that appropriate statistical tools were used for data analysis.

**Comment**

Is the manuscript presented in an intelligible fashion and written in standard English?

PLOS ONE does not copyedit accepted manuscripts, so the language in submitted articles must be clear, correct, and unambiguous. Any typographical or grammatical errors should be corrected at revision, so please note any specific errors here.

Reviewer #1: No

Reviewer #2: No

**Response**

Typographical and grammatical errors have been corrected.

**Comments**

Introduction
1. 3rd paragraph, 4th line: In this regard, Nigeria aimed to vaccin'ate' 40% of its over 200 million people

**Response**

Appropriate correction has been made.

2. 3rd paragraph, 9th line: 'population) are fully vaccinated'. Include what full vaccination means, two, three or four doses?

**Response**

Full vaccination has been defined in the sentence.

3. 4th paragraph, 4th line: It is however important to determine the antibody responses to vaccinated and unvaccinated persons in 'order' to determine possibility of previous SARS-CoV-2 exposure or protection

**Response**

Appropriate correction has been made.

4. 4th paragraph, 7th line : 'S and N proteins of SARS-CoV-2', kindly include full meanings of N and S proteins

**Response**
S and N have been written in full.

Methodology/Materials and Methods
1. Not clear how selection of participant was done
2. How did the authors get details of the vaccinations received by enrolled participants?

**Response**

1. Some participants were consecutively enrolled from individuals coming for voluntary COVID-19 screening at the Biorepository Laboratory, College of Medicine, University of Ibadan. The laboratory is located in Ibadan North Local Government Area of Oyo State, Nigeria. Other participants were randomly enrolled from the general populace in Ibadan North Local Government Area of Oyo State, Nigeria.
2. Details of the type of vaccine received was obtained from the vaccination card.

3. How did authors verify previous RT-PCR tests done by participants for COVID-19

**Response**

The results of all the participants who had RT-PCR tests were verified from the laboratory database. However, we could not verify the responses of other participants who claimed not to have undergone any screening test for SARS-CoV-2 infection. A structured questionnaire was used to obtain this information.

4. Consider rephrasing sentence to make it clearer if authors tested participants for SARS-CoV-2 RT-PCR: Participants with past SARS-CoV-2 infection history and those without were those confirmed positive or otherwise, by real-time reverse-transcriptase polymerase-chain reaction (RT-PCR) assay on nasopharyngeal and oropharyngeal swab specimens following the World Health Organization (WHO) guideline.

**Response**

The sentence has been revised as suggested.

Results
1. Authors did not present data on the period between each vaccination (days? Weeks?), this may explain the statement: Unexpectedly, 23.1% and 21.4% of the participants who have had first and second doses respectively, had no protective antibody to SARS-CoV-2 and The only participant who has had the booster dose was seronegative
**Response**

Discussion on this result has been revised.

Discussion
1. 2nd paragraph, line 5: Consider rephrasing the sentence as number of individuals tested should not be equal to the population of a country. There are guidelines on COVID-19 testing based on symptoms and contact tracing.
**Response**

The sentence has been revised appropriately.

2. 3rd paragraph: Consider providing information of the Nigerian situation of molecular diagnostic testing. The testing is free for the community testing approach and only travelers are charges. Molecular testing capacity in Nigeria has expanded significantly across the country and its no longer limited to reference laboratories

**Response**

The sentence has been revised appropriately.
